# ThMn_12_-type phases for magnets with low rare-earth content: Crystal-field analysis of the full magnetization process

**DOI:** 10.1038/s41598-018-21756-5

**Published:** 2018-02-26

**Authors:** I. S. Tereshina, N. V. Kostyuchenko, E. A. Tereshina-Chitrova, Y. Skourski, M. Doerr, I. A. Pelevin, A. K. Zvezdin, M. Paukov, L. Havela, H. Drulis

**Affiliations:** 1 Faculty of Physics, Lomonosov Moscow State University, 119991 Moscow, Russia; 2 Moscow Institute of Physics and Technology, Dolgoprudny, Moscow region, 9 Institutsky Per., Dolgoprudny, 141700 Russia; 3 Institut für Festkörperphysik, Technische Universität Wien, Vienna, Austria; 4 Institute of Physics CAS, Na Slovance 2, 18221 Prague, Czech Republic; 5 Hochfeld-Magnetlabor Dresden (HLD), Helmholtz-Zentrum Dresden-Rossendorf, D-01314 Dresden, Germany; 6 Technische Universität Dresden, D-01062 Dresden, Germany; 7 Baikov Institute of Metallurgy and Materials Science RAS, 119991 Moscow, Russia; 8 A. M. Prokhorov General Physics Institute of Russian Academy of Sciences, Moscow, 38 Vavilov Str., 119991 Russia; 9 National Research University Higher School of Economics, Myasnitskaya 20, Moscow, 101000 Russia; 10 Faculty of Mathematics and Physics, Charles University, Prague, 12116 Czech Republic; 11 Immanuel Kant Baltic Federal University, Kaliningrad, 236016 Russia; 12 Institute of Low Temperature and Structure Research, Polish Academy of Sciences, 50-950 Wroclaw, Poland

## Abstract

Rare-earth (R)-iron alloys are a backbone of permanent magnets. Recent increase in price of rare earths has pushed the industry to seek ways to reduce the R-content in the hard magnetic materials. For this reason strong magnets with the ThMn_12_ type of structure came into focus. Functional properties of R(Fe,T)_12_ (T-element stabilizes the structure) compounds or their interstitially modified derivatives, R(Fe,T)_12_-X (X is an atom of hydrogen or nitrogen) are determined by the crystal-electric-field (CEF) and exchange interaction (EI) parameters. We have calculated the parameters using high-field magnetization data. We choose the ferrimagnetic Tm-containing compounds, which are most sensitive to magnetic field and demonstrate that TmFe_11_Ti-H reaches the ferromagnetic state in the magnetic field of 52 T. Knowledge of exact CEF and EI parameters and their variation in the compounds modified by the interstitial atoms is a cornerstone of the quest for hard magnetic materials with low rare-earth content.

## Introduction

High demand for commercially viable high-energy permanent magnets stimulates active search for new prospective materials^1^. New here also stands for “well-forgotten” old materials, which can now be upgraded (by elements substitution, tuning of composition, interstitial modifications, etc.) using deeper understanding of fundamental magnetic properties, stemming from modern ideas and techniques. It is the reason why the attention was recently returned to the compounds with the tetragonal ThMn_12_-type of crystal structure for production of rare-earth-lean or rare-earth-free magnets^2–7^. The iron-rich intermetallics RFe_11_Ti, which demonstrate strong uniaxial magnetic anisotropy of the iron sublattice (with the first magnetocrystalline anisotropy constant (MCA) *K*_1_ = 0.89 MJ/m^3^ at room temperature for YFe_11_Ti)^8,9^, are a good example. Interstitial modification of RFe_11_Ti (with magnetic or non-magnetic rare-earth R ions) affect dramatically and usually positively main magnetic characteristics of the compounds such as Curie temperature (*T*_c_), saturation magnetization (*M*_S_), and the first MCA (K_1_)^10–12^.

**Figure 1:**
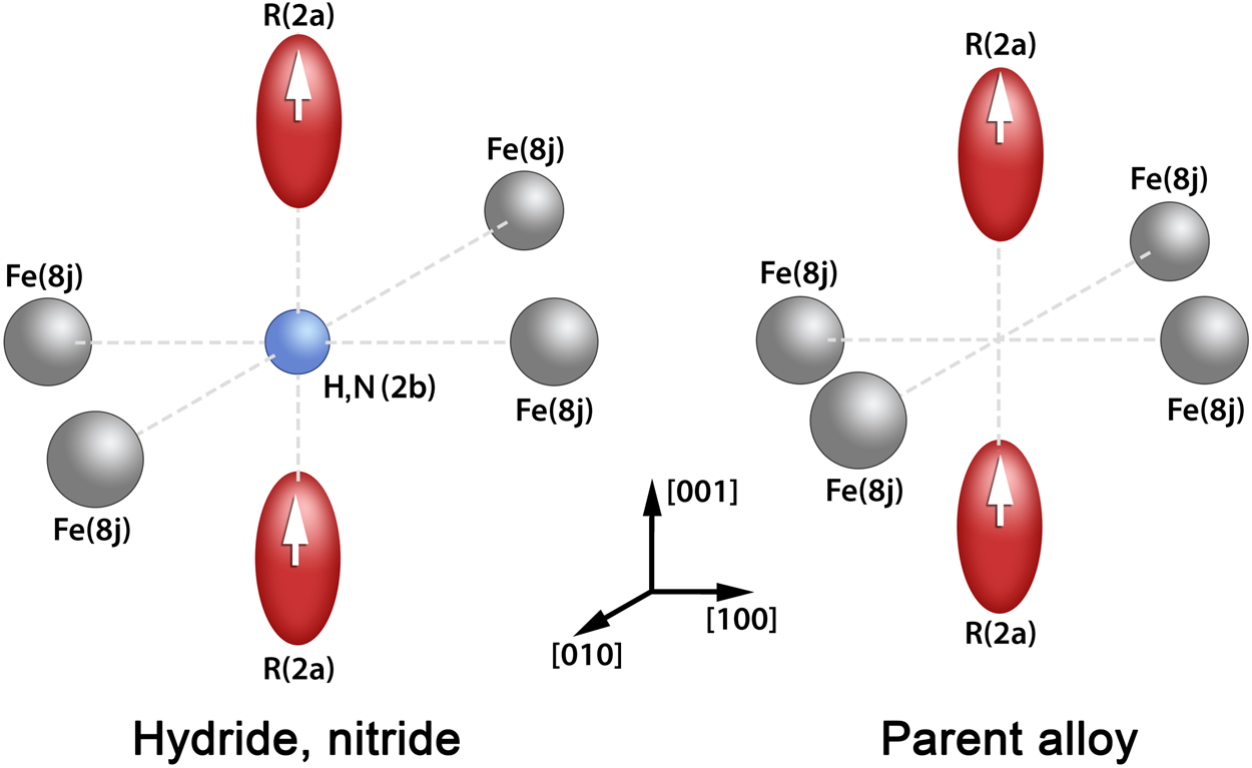
Schematic diagram representing position of hydrogen or nitrogen (arrows indicate the direction of magnetic moments for rare-earth atoms) in RFe_11_Ti-H(N) compounds: left - for the hydride and nitride, right - for the parent alloy.

The crystal lattice of RFe_11_Ti contains a single 2*a* site (point symmetry 4/*mmm*) for the rare-earth atom and three non-equivalent Fe sites (8*i*, 8*j* and 8*f*). Notice that the 8*i* site is populated by a mixture of Fe and Ti^9^. In the case of interstitial modification by hydrogenation or nitrogenation, the H or N atoms occupy a single 2*b* site (see Fig. 1)^9,10^.

Rare earths and iron form two magnetic sublattices, which are either ferromagnetically ordered (co-aligned) in case of light rare-earths or ferrimagnetically aligned (anti-aligned) for heavy rare earths. The rather simple crystal structure of R(Fe,T)_12_ or R(Fe,T)_12_-X streamlines theoretical calculations of the CEF (CEF is the crystalline-electric-field acting on the rare-earth ion) and exchange interaction parameters which can be acquired by analyzing experimental magnetization curves obtained using standard techniques in steady magnetic fields. The examples of the crystal-field analysis can be found in literature for the parent RFe_11_Ti compounds^13–16^, as well as for the hydrided^16,17^ and nitrided^18^ series. Unfortunately, literature data show rather scattered values even within the same series, thus calling for a reliable solution. In order to obtain true CEF and exchange parameters, high magnetic fields should be employed. New experimental techniques allow determination of magnetization in high pulsed magnetic fields up to and above 60 T (with the maximum of 100 T)^19^. Such magnetic fields enable execution of a full magnetization process (i.e. magnetization all the way up to the forced-ferromagnetic state) in ferrimagnets^20^. A particular advantage is to perform such experiments using thulium compounds, since Tm has the Landé factor closest to unity, which allows reaching the ferromagnetic state in relatively weak magnetic fields^21,22^. The second-order CEF parameter at the 2*a* rare-earth site is negative and Tm^3+^ having a positive second-order Stevens’ coefficient α_J_ strengthens the uniaxial anisotropy of the Fe sublattice. Forced-ferromagnetic state can be reached faster (in lower field) if the sample is magnetized along the easy magnetization direction (EMD). Moreover, hydrogen atoms introduced into the crystal lattice of the sample may in general reduce the R-Fe intersublattice exchange interaction thus lowering the field, at which a compound becomes ferromagnetic^22,23^. The purpose of this paper is to calculate to high accuracy the fundamental CEF and exchange interaction parameters in the TmFe_11_Ti and TmFe_11_TiH single crystals from the high-field magnetization measurements and to demonstrate how to control these parameters by modification of the structure with light interstitial elements.

## Experimental details

Polycrystalline TmFe_11_Ti samples were prepared by arc melting of 99.95% pure elements under an argon atmosphere. The ingots were re-melted several times and then heated and cooled slowly in a resistance furnace. Needle-like single crystals (0.7 mm long) were extracted from the ingot and checked by the X-ray Laue technique. Using gentle H_2_-gas hydrogenation procedure, single-crystallinity was preserved upon hydrogenation. Several samples of the TmFe_11_Ti-H system were obtained with different hydrogen concentration close to the upper limit of hydrogen absorption for these materials. The amount of absorbed hydrogen in TmFe_11_TiH_*x*_ (*x* ≈ 0.9, 1 and 1.1) was determined with an accuracy of ±0.02 from the hydrogen pressure change in the calibrated reactor chamber after finishing the reaction. The nitride TmFe_11_TiN_x-δ_ was formed by blowing high-purity nitrogen gas at atmospheric pressure through fine powder samples (grains size less than 10 µm) at 500 °C for 4 h. The nitrogen content was estimated by determining the weight difference of the sample before and after the nitrogenation procedure. The amount of absorbed N_2_ was 1 ± δ atoms per RFe_11_Ti formula unit (δ ≈ 0.05). The nitrided powder was fixed in epoxy resin in a magnetic field of 10 kOe to form aligned samples of cylindrical shape. Powder X-ray diffraction (XRD) analysis was used to determine the structure both of the parent compound and its hydrides and the nitride.

Magnetization measurements were performed using a pulsed-field induction magnetometer at the Dresden High Magnetic Field Laboratory. The maximum field was equal to 60 T and the total pulse duration was 25 ms^19^. Magnetization study in steady magnetic fields was done using a standard PPMS 14 T magnetometer (Quantum Design, USA).

## Results and Discussion

RFe_11_Ti absorb a very small amount of hydrogen (a maximum of 1–1.1 at./f.u.) in contrast to e.g. R_2_Fe_14_B and R_2_Fe_17_ compounds, where the amount of absorbed hydrogen can reach 5 at./f.u.^11^. XRD study showed that TmFe_11_TiH_*x*_ (*x* = 0, 0.9, 1 and 1.1) and TmFe_11_TiN_*x*_ (*x* = 1) retained the tetragonal ThMn_12_ type (space group *I*4/*mmm* and Z = 2) of crystal structure after hydrogenation and nitrogenation. Lattice parameters are shown in Table 1.

**Table 1:** Unit cell parameters of TmFe_11_Ti-(H,N).

Compound	*a*, nm	*c*, nm	*V*, nm^3^	Δ*V/V*,%
TmFe_11_Ti	8.4655	4.7794	342.5	0
TmFe_11_TiH_0.9_	8.5013	4.7790	345.4	0.8
TmFe_11_TiH_1_	8.5053	4.7789	345.7	0.9
TmFe_11_TiH_1.1_	8.5093	4.7789	346.0	1
TmFe_11_TiN_1_	8.5542	4.8157	352.4	2.8

One can see that hydrogenation leads to an increase of the *a* parameter while *c* slightly decreases. Volume expansion of the hydrides does not exceed 1%. Nitrogenation increases the relative unit cell volume Δ*V/V* by 3%.

**Figure 2:**
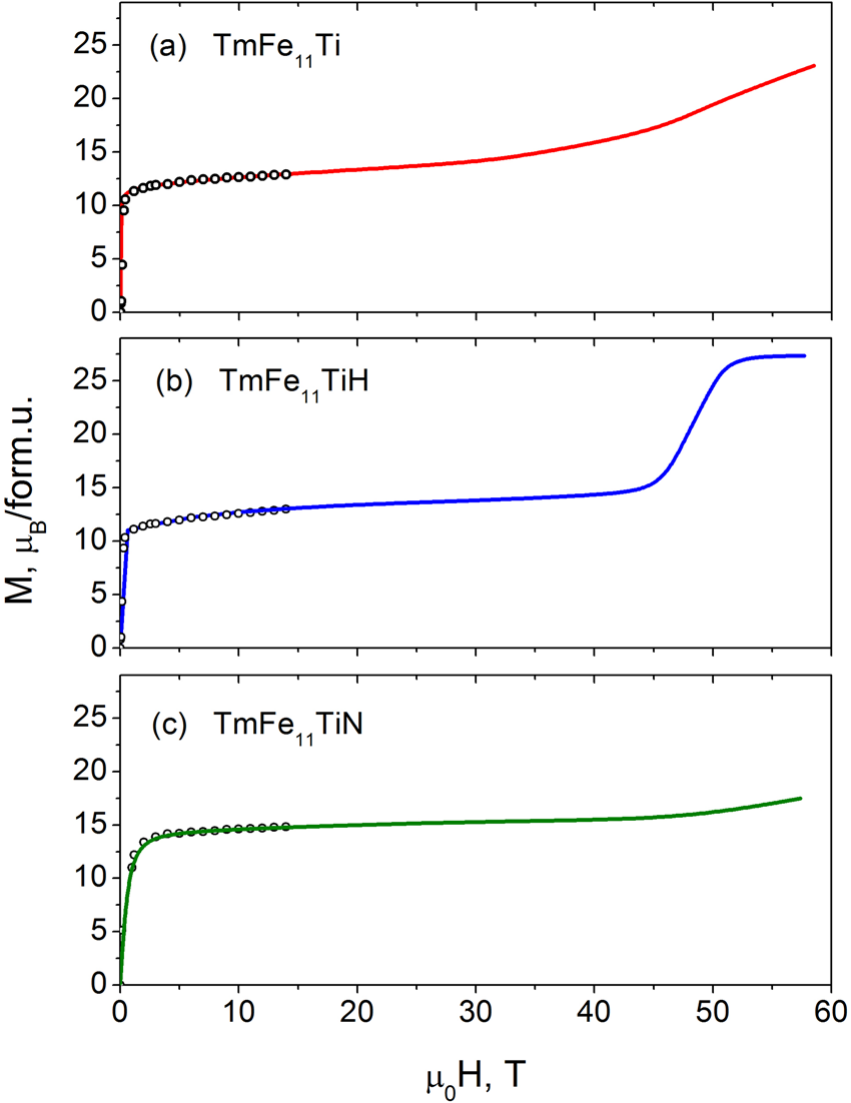
Magnetization curves of TmFe_11_Ti, TmFe_11_TiH and TmFe_11_TiN measured at 4.2 K in pulsed fields applied along the [001] axis (dots represent measurements in steady magnetic fields).

Figure 2 shows the magnetization curves at 4.2 K for the single crystalline TmFe_11_Ti and TmFe_11_TiH samples and for the aligned powder sample TmFe_11_TiN in magnetic fields applied along the [001] axis. It is seen, that the ferromagnetic state is reached in the magnetic field of 52 T for TmFe_11_TiH. Magnetic fields exceeding 60 T are needed to observe a ferromagnetic state both in the parent and in the nitrided compounds. It was shown earlier that Tm_2_Fe_17_^24^ and Tm_2_Fe_14_B^21^ reach the ferromagnetic state in fields exceeding 80 T. It is clear that nitrogenation enhances the intersublattice exchange interaction in TmFe_11_Ti.

**Figure 3:**
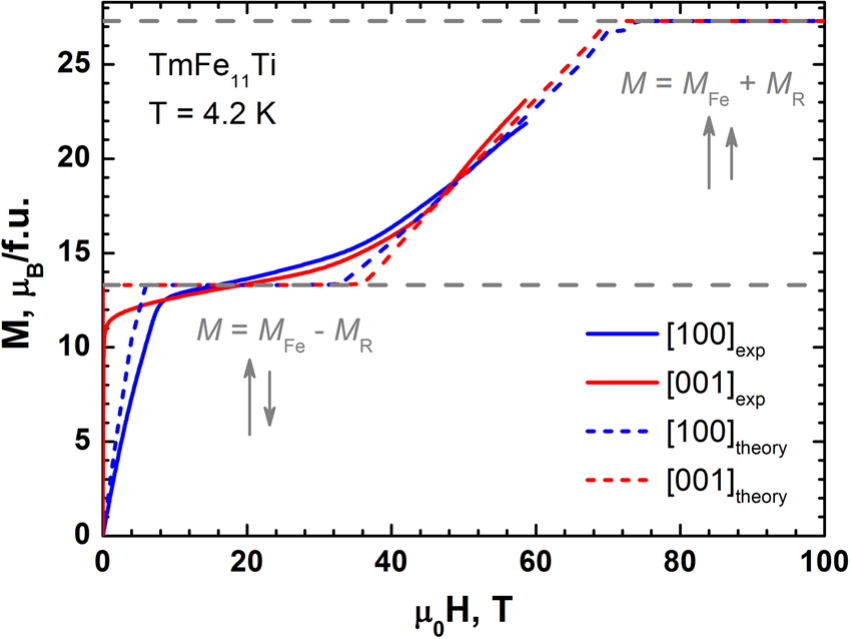
Theoretical (dashed lines) and experimental (solid lines) magnetization curves obtained for the TmFe_11_Ti single crystal in magnetic fields applied along the [001] and [100] axis at 4.2 K.

**Figure 4:**
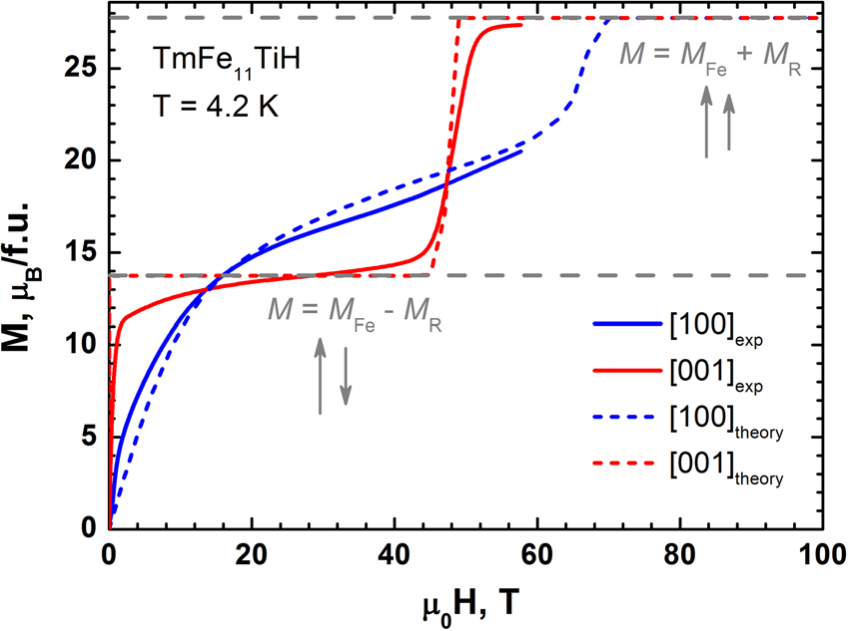
Theoretical (dashed lines) and experimental (solid lines) magnetization curves obtained for the TmFe_11_TiH single crystal in magnetic fields applied along the [001] and [100] axis at 4.2 K.

Figures 3 and 4 demonstrate the magnetization curves of TmFe_11_Ti and TmFe_11_TiH at 4.2 K taken along crystallographic directions [001], [100] and [110].

While the four-fold symmetry axis *c* ([001]) is the easy magnetization direction, the two basal-plane curves [100] and [110] (not shown) measured along the hard magnetization directions look practically identical due to a weak anisotropy within the basal plane. In the parent TmFe_11_Ti compound, the basal-plane magnetization curve crosses the *c*-axis magnetization at about 9 T, running above it close together in the interval from 9 T to 60 T. The TmFe_11_TiH hydride shows only one step on the magnetization curve at 45 T. As a result, TmFe_11_TiH proceeds from the ferrimagnetic state (where the total magnetization *M*s = *M*_Fe_ − *M*_R_ = 13.8 µ_B_/f.u) directly to the forced ferromagnetic state, with *M*_S_ = *M*_Fe_ + *M*_R_ = 27.8 µ_B_/f.u.

The magnetic behavior of the TmFe_11_Ti-H system was described through quantum theory analysis, using a two-sublattice (rare-earth and iron sublattices) approximation for the magnetic structure and taking into account the exchange and CEF interactions. The method of theoretical investigation is essentially the same as described in refs^15,25–27^. It is a general method of calculating magnetic properties in the R-M-X systems, where R and M are the 4f and 3d-transition metals and X is a non-magnetic element such as boron. In brief, the total free energy in this model has the form

1 $$\begin{array}{rcl}F & = & -N{k}_{B}T\,\mathrm{log}\,Z-{M}_{Fe}({H}_{x}\,\sin \,\theta \,\cos \,\varphi +{H}_{y}\,\sin \,\theta \,\sin \,\varphi +{H}_{z}\,\cos \,\theta )\\  &  & +{K}_{1}\,{\sin }^{2}\theta +{K}_{2}\,{\sin }^{4}\theta .\end{array}$$

where ${\rm{Z}}={\sum }_{{\rm{n}}}\exp (\frac{-{{\rm{E}}}_{{\rm{n}}}}{{{\rm{k}}}_{{\rm{B}}}{\rm{T}}})$, E_n_ is the energy of the n-th level of the R ion. The second term of Eq. (1) represents the energy of the iron sublattice, the angles *θ* and *φ* are polar coordinates of the iron sublattice magnetization with respect to the main crystallographic directions ([001] and [100]), *H* = (*H*_x_*, H*_y_*, H*_z_) is the external magnetic field, *K*_1_ and *K*_2_ are the anisotropy constants of the second and fourth order. Numerical values of the polar coordinates *Θ* and *φ* are obtained by minimizing the total free energy at given conditions (temperature, direction and magnitude of external magnetic field).

Hamiltonian of the rare-earth ion is Hermitian matrix with the dimension (2*J* + 1) × (2*J* + 1) (*J* is the total angular momentum of the ground Tm^3+^ multiplet) and is given by:

2 $$H={H}_{CF}+{g}_{J}{\mu }_{B}{\boldsymbol{J}}({{\boldsymbol{H}}}_{ex}+{\boldsymbol{H}})$$

where *g*_J_ is the Landé factor, *H*_ex_ is the exchange field.

The crystal-field Hamiltonian is:

3 $${H}_{CF}={B}_{0}^{2}{C}_{0}^{2}+{B}_{0}^{4}{C}_{0}^{4}+{B}_{0}^{6}{C}_{0}^{6}+{B}_{4}^{4}({C}_{-4}^{4}+{C}_{4}^{4})+{B}_{4}^{6}({C}_{-4}^{6}+{C}_{4}^{6})$$

with the crystal-field parameters ${{\rm{B}}}_{{\rm{q}}}^{{\rm{k}}}$ and single-electron irreducible tensor operators ${{\rm{C}}}_{{\rm{q}}}^{{\rm{k}}}={\sum }_{{\rm{i}}}{{\rm{C}}}_{{\rm{q}}}^{{\rm{k}}}({\rm{i}})$.

The magnetization behavior of the system is obtained by using the following expression

4 $${M}_{\alpha }=-\frac{\partial F}{\partial {H}_{\alpha }},\quad \alpha =x,\,y,\,z$$

The total magnetization of the parent TmFe_11_Ti was estimated as *M*s = *M*_Fe_ − *M*_R_ = 13.3 µ_B_/f.u and this value is in good agreement with experiment. Estimated value of magnetization in the ferromagnetic state is *M*_S_ = *M*_Fe_ + *M*_R_ = 27.3 µ_B_/f.u. The exchange fields *H*_ex_ for TmFe_11_Ti and TmFe_11_TiH_1_ obtained by fitting our experimental magnetization data are 50.8 and 47.5 T, respectively. The CEF parameters for both compounds are presented in Table 2.

**Table 2:** CEF (in cm^−1^) and exchange (in T) parameters for TmFe_11_Ti and TmFe_11_TiH obtained by fitting the experimental magnetization data in the present work.

Compound	${{\boldsymbol{B}}}_{{\bf{0}}}^{{\bf{2}}}$	${{\boldsymbol{B}}}_{{\bf{0}}}^{{\bf{4}}}$	${{\boldsymbol{B}}}_{{\bf{0}}}^{{\bf{6}}}$	${{\boldsymbol{B}}}_{{\bf{4}}}^{{\bf{4}}}$	${{\boldsymbol{B}}}_{{\bf{4}}}^{{\bf{6}}}$	*H*_ex_
TmFe_11_Ti	−17.1	−2.21	43.92	−23.98	0	50.8
TmFe_11_TiH	−50.2	−40.02	43.92	−23.98	0	47.5

CEF and exchange parameters of the parent TmFe_11_Ti were already obtained in refs^13,28^ in fields up to 30 T. Our investigations in the magnetic fields up to 60 T allowed us to obtain redefined values of these parameters, especially of ${B}_{0}^{2},\,{B}_{0}^{4},\,{B}_{0}^{6}$ and *H*_ex_. The calculated CEF and exchange parameters for different hydrogenated samples are very close to each other because of the not large span of hydrogen content in the studied samples (x ranges between 0.9 and 1.1). However, there is a significant difference in the values of parameters between parent and hydrogenated compounds, especially, of ${B}_{0}^{2},\,{B}_{0}^{4}$. The parameters for the TmFe_11_TiH hydride are obtained for the first time.

The changes in the ${B}_{0}^{2}$ and exchange parameter after hydrogenation of TmFe_11_Ti attract a special attention. ${B}_{0}^{2}$ increases almost by a factor of 3 leading to a significant strengthening of uniaxial anisotropy^29,30^. The exchange field *H*_ex_ slightly decreases from 50.8 to 47.5 T. Note, a slight increase in the R-Fe exchange interaction was reported previously for HoFe_11_TiH^17^, for which the studies were performed in the magnetic field much lower than the field of the transition from ferri- to the ferromagnetic state. This *H*_ex_ parameter is responsible for reaching of the forced-ferromagnetic state in fields lower than 60 T^22^. Using the obtained full set of parameters we were able to calculate theoretical magnetization curves up to 100 T: it is especially important for the parent compound where the transition to the ferromagnetic state occurs in the magnetic fields (near 70 T) slightly exceeding the applied 60 T limit.

## Summary

We have studied the magnetic properties of TmFe_11_Ti-X hydrides and a nitride in high magnetic fields. The main feature of our work is that in the case of a hydrided TmFe_11_Ti we were able to conduct theoretical analysis using a full magnetization process obtained experimentally. We also established that introduction of 1 H at./f.u. into TmFe_11_Ti weakens the intersublattice exchange despite the increasing magnetic moment on Fe atoms or, in other words, despite the strengthening of the Fe sublattice. Based on the results obtained we can provide the following recommendations. The obtained second-order crystal-field parameter ${B}_{0}^{2}$ for RFe_11_Ti is negative and small, but its value can be easily controlled by changing environment of the rare-earth ion with the aid of interstitial (Fig. 1) and by substitution atoms^9^. Here, we solve a direct problem, namely, we determine parameters of the crystal and exchange field from experimental magnetization curves using single-crystalline samples. It will also be possible to solve inverse problem^16^ since R-Fe crystal field parameters do not change significantly within one series of compounds with various Rs. It will enable design and simulation (see e.g. studies predicting new materials)^31,32^ of compounds with desired magnetic properties when we substitute an expensive rare-earth by cheaper R ions (for example, cerium) and/or other elements (for example, zirconium). Indeed, zirconium is already widely used for R-lean magnetic materials^5^. Promising magnetic characteristics were demonstrated e.g. for R(Fe,T)_12_ (where R = Nd) compounds interstitially modified by nitrogen^3,18^. This gives hope that strong permanent magnets with low rare-earth content may soon become a reality.
